# Determination of Metals in Natural Waters by Inductively Coupled Plasma Optical Emission Spectroscopy after Preconcentration on Silica Sequentially Coated with Layers of Polyhexamethylene Guanidinium and Sulphonated Nitrosonaphthols

**DOI:** 10.1155/2019/1467631

**Published:** 2019-07-01

**Authors:** Svetlana L. Didukh-Shadrina, Vladimir N. Losev, Alexandr Samoilo, Anatoliy К. Trofimchuk, Pavel N. Nesterenko

**Affiliations:** ^1^Scientific Research Engineering Centre “Kristall”, Siberian Federal University, Krasnoyarsk 660041, Russia; ^2^Taras Shevchenko National University of Kyiv, Kyiv 01601, Ukraine; ^3^Chemistry Department, Lomonosov Moscow State University, Moscow 119991, Russia

## Abstract

A series of complexing adsorbents is prepared by coating silica particles with linear polyhexamethylene guanidinium (PHMG) chloride followed by saturation with a number of sulphonated nitrosonaphthols reagents electrostatically retained by positively charged polymer layer. PHMG coated silica is hydrolytically stable even during treatment with 6 M HCl heated up to 50 °C. The adsorption of 1-nitroso-2-naphthol-3,6-disulfonic acid (nitroso-R-salt), 2-nitroso-1-naphthol-4-sulfonic acid (nitroso-N-salt), and 2-nitroso-1-naphthol-3,6-disulfonic acid (nitroso-K-salt) on PHMG modified silica was studied. The effective immobilisation of sulphonated nitrosonaphthols was achieved in the range of pH of 3 - 8, while the adsorption of the monosulphonated reagent (nitroso-N-salt) is twice as high as the disulphonated analogues (nitroso-R-salt and nitroso-K-salt). The adsorption of Cu(II), Fe(III), Co(II), Ni(II), Al(III), Zn(II), Pb(II), Mn(II), and Cr(III) on prepared complexing adsorbents under static and dynamic conditions was studied as a function of time, pH, sample volume, and presence of interfering ions. Metal ions can be desorbed by using 1 M HCl or 1 M HNO_3_. The preconcentration factors of metals under optimized conditions are varied from 20 to 80. The developed method was used for the preconcentration of trace metals from natural waters followed by ICP-OES determination. The sub-ppb limits of detection of metals are achieved.

## 1. Introduction

The determination of metal ions in natural waters at low concentration level remains one of the most important tasks in environmental monitoring. The application of various spectrometric techniques such as flame atomic-absorption spectrometry (FAAS), graphite furnace atomic spectrometry (GFAAS), inductively coupled plasma optical emission spectrometry (ICP-OES), and mass-spectrometry (ICP-MS) has been reported for multielement determination of metals [1–3]. The choice of right specific analytical method depends on concentration of analytes and number of metals to be detected in the sample.

However, group determination of metals at trace level in complex matrices requires sample pretreatment with the purpose of preconcentration of target analytes and/or elimination of undesirable matrix effects.

The most efficient way for the preconcentration of traces of transition and heavy metals from waters is solid phase extraction (SPE) with various types of adsorbents including chemically modified silica, nanocarbon containing materials, magnetic particles, chitosan based, and many others [2, 4–9]. Despite the wide range of adsorbents reported for the preconcentration of trace elements from natural waters, the development of new highly selective adsorbents combining simplicity of synthesis with low-cost of the production remains an important task. The selectivity of the adsorbents to the analytes is primarily determined by the nature of the functional group chemically or noncovalently fixed on the surface of solid matrices [2, 5, 8, 9].

Nitrosonaphthols and their structural analogues have attracted attention as selective ligands for the spectrophotometric and chromatographic determination of transition metals [10–12] and for the preparation of chelating adsorbents. The chelating adsorbents can be prepared by covalent attachment of 1-nitroso-2-naphthol [13] and 2-nitroso-1-naphthol [14] to macroreticular poly(styrene-divinylbenzene) (PS-DVB) resins as well as 1-nitroso-2-naphthol to silica gel [15]. The possibility of the preconcentration of V(V), Cr(III), Mn(II), Fe(III), Co(II), Ni(II), Cu(II), Zn(II), Hg(II), Pb(II), Cd(II), Zn(II), Al(III), Pd(II), and U(VI) from water solutions is studied. The covalent grafting of this type of ligands to a suitable matrix is a complex task, so physical adsorption or impregnation can be used as alternative for their immobilisation of nitrosonaphthols.

The first successful application of hydrophobic PS-DVB resin impregnated with neutral 1-nitroso-2-naphthol reagent for the preconcentration of cobalt is reported by Kubo et al. in 1977 [16]. The high preconcentration factor (PF) of 67 was achieved for cobalt in this work. Aydin and Soylak used PS-DVB resin MCI GEL CHP20P impregnated with structurally similar 2-nitroso-1-naphthol reagent as adsorbent for preconcentration of Th(IV), Ti(IV), Fe(III), Pb(II), and Cr(III) prior to ICP-MS determination [17]. The silica based adsorbent prepared by impregnation with 1-nitroso-2-naphthol was also used for the preconcentration of cobalt, while a higher PF of 100 was achieved [18].

There are few methods of using electrostatic interactions for the immobilisation of sulphonated nitrosonaphthols such as 1-nitroso-2-naphthol-3,6-disulfonic acid (nitroso-R-salt, NRS) and 2-nitroso-1-naphthol-4-sulfonic acid (nitroso-N-salt, NNS). The simple way is based on a conversion of PS-DVB strong base anion-exchange resins into NRS form. The modification of anion-exchange resins Dowex 1 [19–22], Amberlite IRA-402, and IRA-958 [23] has been reported.

A few approaches for the preparation of chelating adsorbents containing metal-selective nitrosonaphthol functional groups have been described in the literature [24–27]. Kocjan et al. prepared chelating adsorbent by impregnation of silica with ion-pair composed of a mixture of tricaprylylmethylammonium and trioctylmethylammonium cations (Aliquat 336) and NRS or NNS [24, 25]. NRS containing adsorbent was used for the chromatographic separation of Co(II) <Fe(III), Cu(II) <Ni(II) <Cd(II), Zn(II), and Mn(II) with step gradient of perchloric acid as elution. A baseline separation of Co(II), Fe(III), Ni(II), and Ca(II) was obtained on a column packed with NNS containing adsorbent [24, 25]. The authors also reported selectivity for NNS adsorbent estimated as adsorption capacities for different metals. At pH 6.0 the following selectivity was observed: Co(II) (14.2) > Cu(II) (6.9) > Ni(II) (6.4) > Cr(III) (5.9) >Pb(II) (5.1) > Zn(II) ≈ Al(III) (4.2) > Cd(II) (3.6) >Mn (2.6), and no adsorption was noted for Na(I), K(I), Ca(II), and Mg(II). It was also noted that NNS forms more stable ion-pairs with Aliquat 336 than the more hydrophilic NRS.

Later, this research group used the same approach for the preparation of chelating adsorbents by coating of hydrophobic octylsilica and octadecyl silica substrates with ion-pairs formed by Aliquat 336 and NNS [24, 25]. A complete separation of Mg(II) < Cu(II) < Fe(III) < Co(II) was obtained with gradient elution using perchloric acid as eluent.

**Scheme 1:**
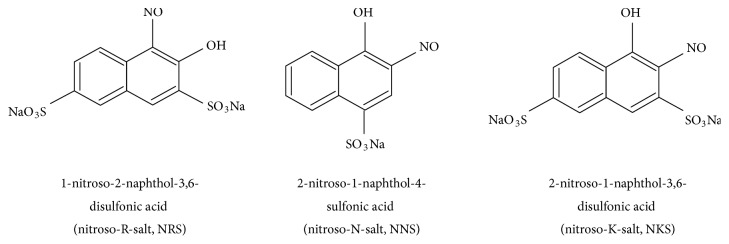


Recently, two-step modification of silica surface was proposed for the immobilisation of negatively charged chelating reagents. At the first step bare silica is coated with linear polyhexamethylene guanidine [-(CH_2_)_6_-NH-C(=NH_2_^+^Cl^−^)-NH-]_n_ (PHMG) to obtain an intermediate SiO_2_-PHMG with positively charged surface. The prepared product is treated with solution of negatively charged organic reagents, such as sulphonated nitrosonaphthols, which are strongly retained due to electrostatic interactions [26, 27]. The resulting adsorbents had a three-layer structure formed by layers of negatively charged silanol groups and positively charged protonated PHMG polymer layer and outer layer of negatively charged chelating reagents. In this work three nitrosonaphthols containing one (nitroso-N-salt, NNS) or two sulpho (nitroso-K-salt, NKS and nitroso-R-salt, NRS) groups in their molecules (see Scheme 1) were used for the preparation of adsorbents by modification of SiO_2_-PHMG. The PHMG coated silica modified with 1-nitroso-2-naphthol-3,6-disulfonic acid (nitroso-R-salt, NRS) has been successfully used for the preconcentration and determination of Pd(II) and Co(II) [28, 29].

The aim of the present work is connected with preparation of three new silica based adsorbents by consecutive treatment of the surface with PHMG and sulphonated nitrosonaphthols and investigation of their adsorption properties towards a group of transition metals cations with focus on the development of ICP-OES method of natural waters analysis. The presence of oppositely charged layers at the surface of adsorbents and chelating properties of residual guanidinium groups in PHMG can influence both selectivity and kinetics of metals adsorption as indicated in the literature [30, 31].

## 2. Materials and Methods

### 2.1. Reagents

All reagents used in this work had analytical grade. Standard stock solutions of Cu(II), Fe(III), Co(II), Ni(II), Al(III), Zn(II), Pb(II), Mn(II), and Cr(III) (100 mg L^−1^) were prepared by dissolving the corresponding salts in 1 mol L^−1^ nitric acid and further diluted in double distilled deionized water on daily basis. 1 M HNO_3_, 1 M NaOH, 0.1 M acetate–acetic acid buffer (pH 4.0 – 6.3), 0.5 M ammonia–ammonium acetate buffer (pH 6.5 – 7.0), and 0.1 M ammonia–ammonium chloride buffer (pH 8.0 – 8.5) were used for pH adjustments. The silica gel Silokhrom S-120 (Reakhim, Stavropol, Russia) having particle size 0.1 – 0.2 mm, specific surface area of ~120 m^2^g^−1^, and pores of average diameter 45 nm was used as a matrix for the preparation of adsorbents. Polyhexamethylene guanidine chloride (PHMG, 95% pure) was supplied by the Institute of Eco-Technological Problems (Moscow, Russia). 1-Nitroso-2-naphthol-3,6-disulfonic acid (nitroso-R-salt, NRS), 2-nitroso-1-naphthol-4-sulfonic acid (nitroso-N-salt, NNS), and 2-nitroso-1-naphthol-3,6-disulfonic acid (nitroso-K-salt, NKS) were obtained from Sigma-Aldrich (St. Louis, MO, USA).

### 2.2. Equipment

ICP-OES spectrometer Optima 5300DV (Perkin-Elmer) with a cross-flow nebulizer and a Ryton Scott chamber was used for the detection of metal ions. Elemental measurements were made in axial view mode using wavelengths and operating parameters recommended for the spectrometer, which are presented in Table 1.

The pH values were adjusted using a Seven Easy S20 digital pH meter (Mettler-Toledo, Switzerland) calibrated using four standard buffer solutions of pH 1.65, 4.01, 7.00, and 9.18. UV-Vis spectra were recorded with Cary 100 spectrophotometer (Varian, Australia). A MasterflexL/S peristaltic pump (Thermo Fisher Scientific, USA) was used for pumping solutions through a glass minicolumn (30 x 3 mm I.D.) containing 0.1 g of the sorbent.

Thermogravimetric analysis (TGA) of prepared adsorbents was performed with STA 449 C analyser (Netzsch, Germany) coupled to FTIR spectrometer Nicolet 380 via TGA/FT-IR interface (Thermo Scientific, USA). This instrument can be used for differential scanning calorimetry, thermogravimetry, and analysis of the released gas phase. FTIR spectra are used for measurement of absorbance of gases released from the sample heated in platinum crucible in the temperature range from 30 to 760°С.

Semiempirical method PM3 of computational chemistry software GAMESS was used for the calculation of geometry of the molecules. Molecular parameters are calculated for the most stable conformational forms using geometric parameters and van der Waals atomic radii.

### 2.3. Preparation of Adsorbents

To activate surface silanol groups 15 grams of silica was soaked in diluted solution of NaOH with рН 9.0 for 1 hour and carefully washed by deionised water until neutral pH. Adsorbent SiO_2_–PHMG was obtained by addition of 100 mL of 7.5% solution of PHMG hydrochloride in water to activated silica at rate 1 mL min^−1^ and constant mixing. The prepared adsorbent was washed with deionised water until negative reaction on presence of PHMG in rinsing waters indicated by test reaction with bromphenol blue at pH 11 in presence of sodium dodecylsulfate and chloroform as described [27]. The prepared adsorbent was dried in the air at 70°C.

The immobilisation of sulphonated nitrosonaphthols at the surface of SiO_2_–PHMG was optimised by additional set of experiments. In this study 10 mL of sulphonated nitrosonaphthol with concentration varied from 6.8·10^−6^ М to 1.4·10^−3^ М was added to 0.1 g amounts of SiO_2_–PHMG placed in test tubes and agitated for 5 min. The prepared adsorbents (SiO_2_–PHMG–NRS, SiO_2_–PHMG–NNS, and SiO_2_–PHMG–NКS) were decanted and dried in the air. The adsorption of reagents was calculated from the changes in absorbance of solutions measured spectrophotometrically at 375 nm (NRS), 383 nm (NNS, pH<6.0), 424 nm (NNS, pH>6.0), and 368 nm (NKS) before and after equilibration.

### 2.4. Investigation of Metal Ions Adsorption under Static Conditions

10 mL of solutions containing 0.2 *μ*g mL^−1^ of Cu(II), Fe(III),Co(II), Ni(II), Al(III), Zn(II), Pb(II), Mn(II), and Cr(III) was added to 0.1 g amounts of SiO_2_-PHMG-NRS or SiO_2_-PHMG-NNS. pH of solutions were adjusted to рН 2.0 - 3.0 by addition of concentrated HNO_3_ and to рН 4.0 - 7.0 and pH 7.5 – 9.0 by addition of acetate and ammonium chloride buffers, respectively. The mixture was agitated for 1 - 30 min and then residual concentration of metals in solutions was measured by ICP-OES. Additionally the metals concentrations were measured in 0.1, 1, 2, 4, and 6 М HCl or HNO_3_ solutions used for desorption of adsorbed metals.

### 2.5. Metal Ions Preconcentration in Dynamic Conditions

The adsorption of metal ions in dynamic mode was studied with 3 cm long and 3 mm I.D. chromatographic column containing 0.1 g of SiO_2_-PHMG-NRS or SiO_2_-PHMG-NNS adsorbents with maximum loadings of corresponding reagents. 10 – 100 mL aliquots of 0.2 *μ*g mL^−1^ metal ions solutions with pH varied from 2 to 8 were passed through the column at flow rate 0.5 - 5 mL min^−1^ by using peristaltic pump. The metals were desorbed from the column by pumping through 5 or 10 mL of 1 M HNO_3_. ICP-OES was used for the measurements of metal concentration in the effluents.

The efficacy of adsorbents for isolation of metals was evaluated by using preconcentration factors (PFs). Assuming both quantitative adsorption and quantitative desorption under optimised condition the values of PFs were calculated as ratio of initial sample volume to the minimal volume of the eluent required for desorption of the selected metal ion.

### 2.6. Sample Analysis

100 mL of the sample was filtered through acetyl cellulose membrane filter with pore diameter of 0.1 *μ*m (Vladipore, Vladimir, Russia) and acidified with concentrated HNO_3_ to рН 1.0. To destroy organic substances the samples were boiled for 30 minutes; then pH of cooled samples was adjusted to рН 6.0 and passed through the column at flow rate 1.5 mL min^−1^. Desorption of metals before their determination in effluents was performed as described in Section 2.5.

## 3. Results and Discussion

The prepared adsorbents have three layered structures composed of negatively charged silanol groups, intermediate layer of positively charged complexing polymer PHMG, and outer layer of adsorbed sulphonated reagents. The combined complexing properties are defined by the presence of nitrosonaphthols groups from the electrostatically retained reagents and unshielded guanidinium groups from PHMG layer. The ratio of nitrosonaphthol and guanidinium groups can be regulated through number of sulpho groups in the molecules of sulphonated nitrosonaphthols (one sulfo group in NNS and two in NKS) and variation of reagent loadings. The selectivity of the prepared adsorbents depends also on position of nitroso and naphthol groups in isomeric reagents NRS and NKS. On this reason the adsorption of sulphonated nitrosonaphthols on SiO_2_-PHMG and metal ions on the prepared complexing adsorbents was additionally investigated.

### 3.1. Adsorption of Sulphonated Nitrosonaphthols on SiO_2_-PHMG

**Figure 1:**
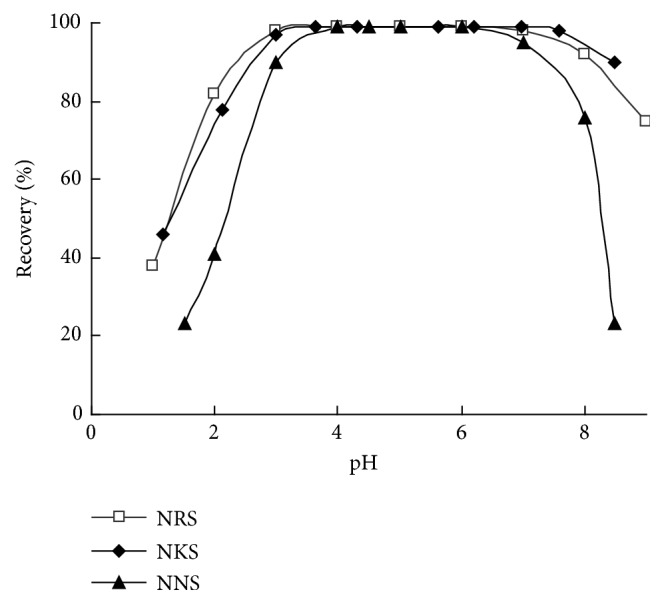
Effect of pH on NRS, NKS, and NNS adsorption on SiO_2_–PHMG as a function of рН. *m*_*ads*_ = 0.1 g, *C*_*О*R_=2.65·10^−5^М, sample volume 10 mL, and shaking time 5 min.

**Figure 2:**
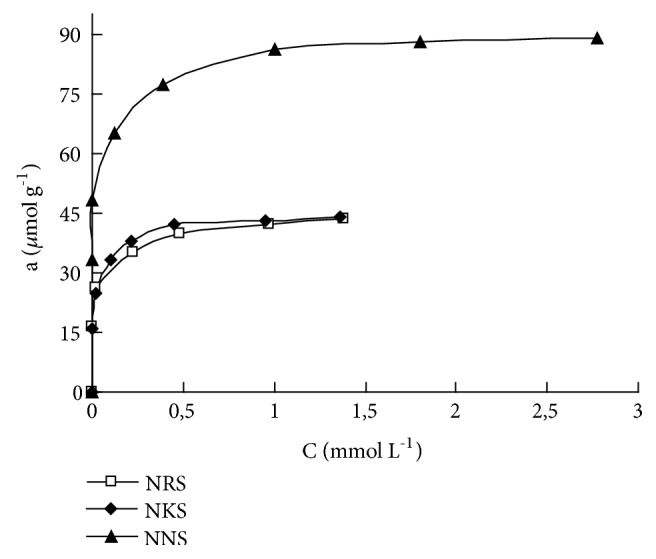
Adsorption isotherms of NRS, NKS, and NNS on SiO_2_–PHMG at pH 5. The other conditions are as shown in Figure 1.

According to TGA data the intermediate product SiO_2_-PHMG contains 1.36% of loaded PHMG; therefore surface concentration of positively charged guanidinium groups can be estimated as 96 *μ*equiv g^−1^ for this adsorbent. It was found that PHMG modified silica binds quantitatively (recovery 98 - 99%) NRS and NKS from aqueous solutions in pH range from 3.0 to 8.0 and NNS in pH range from 4.0 to 7.0 (Figure 1). Maximum adsorption capacity of SiO_2_–PHMG for sulfonated nitrosonaphthols was calculated from isotherms of adsorption, obtained for the saturation of the sorbent surface with reagents at pH 5 (Figure 2). The adsorption equilibrium requires less than 5 min for all nitrosonaphthols. The decrease of adsorbed NRS, NKS, and NNS observed at рН < 2 is associated with protonation of naphthylsulfonic groups in these reagents (*рК*_*а*_ ≤ 2.5) resulting in formation of neutral molecular forms. Obviously, neutral forms of sulphonated nitrosonaphthols have strong electrostatic interactions with surface of SiO_2_–PHMG. Similarly, the adsorption of sulphonated nitrosonaphthols decreases with increase of pH above 7 due to deprotonation of guanidinium groups.

Clearly, adsorption capacity values for sulfonated nitrosonaphthols depend on the charge of the molecules and were equal to 43 *μ*mol g^−1^ for double charged NRS and NKS and 88 *μ*mol g^−1^ for single charged NKS reagent. These are close to the maximum ion-exchange capacity 96* μ*equiv g^−1^ of SiO_2_-PHMG, while only 10% of guanidine groups do not form ion associates with sulphonated nitrosonaphthols. It should be noted that the adsorption capacity value of SiO_2_-PHMG obtained for NNS is twice higher than for NRS and NKS (Figure 2).

According to computational calculations the size of monosulphonated NNS molecule is about 3.98 nm^2^ that is comparable with the value 4.23 nm^2^ calculated for disulphonated NRS molecules; therefore the difference in adsorption capacity values is due to different orientation of the molecules upon the surface of SiO_2_-PHMG. Possibly, NRS and NKS molecules have two interaction points being planar or parallel to the surface orientation, while NNS molecule with one binding point is perpendicular to the surface orientation. The amount of NRS adsorbed on SiO_2_-PHMG is higher than 20 *μ*mol g^−1^ NRS loading reported in the literature for PS-DVB anion-exchange resin [20].

Obviously, sulphonated nitrosonaphthols are retained on the surface SiO_2_–PHMG due to electrostatic interactions between bulky polarisable organic anions and guanidinium groups from PHMG; therefore the concentration of adsorbed reagents can be sensitive to the presence of other anions. The stability of the prepared adsorbents was studied by treatment with concentrated solutions of sodium chloride and hydrochloric and nitric acids. According to the theory of ion-exchange [42] the desorption degree of single charged NNS anion is significantly higher than of double charged NRS and NKS anions for all solutions used for treatment (Tables 2 and 3). It should be noted that a small part of sulphonated nitrosonaphthols is strongly retained by SiO_2_–PHMG and can be desorbed only by two washes with 6 M inorganic acid heated to 50°С.

**Figure 3:**
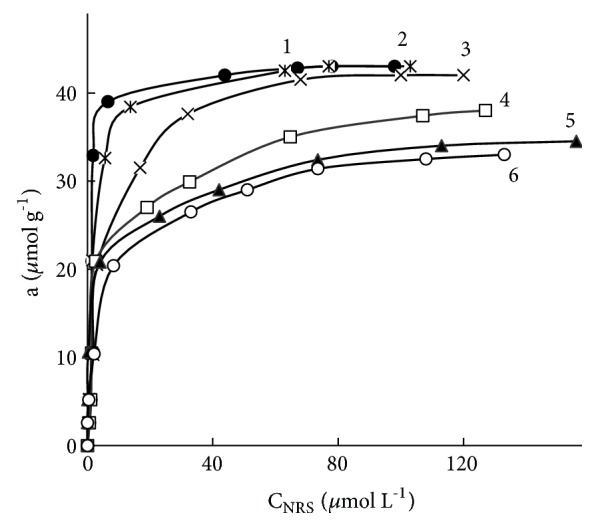
Adsorption isotherms of NRS on SiO_2_–PHMG before (1) and after one (2), two (3), three (4), four (5), and five (6) treatments of SiO_2_–PHMG–NRS with 10 mL of hot (50°С) 6 М  НСl. Mass of adsorbent-0.1 g, other details of experiments as listed in Figure 1.

Acid treatment can affect the stability of the prepared adsorbents by weakening electrostatic interactions not only between sulphonated nitrosonaphthols and protonated PHMG, but also between PHMG layer and silanols groups from the silica backbone. However, our investigation demonstrated excellent stability of SiO_2_–PHMG.  Figure 3 shows the isotherm of adsorption of NRS on the surface of SiO_2_–PHMG treated few times with hot (50°С) 6 М  НСl. Clearly, as shown in Figure 3 the adsorption of NRS did not change dramatically at least after three such treatments. It means that SiO_2_–PHMG can be reloaded with NRS and reused few times if required.

### 3.2. TGA of SiO_2_–PHMG–NRS and SiO_2_–PHMG–NNS

To identify the decomposition products of adsorbents a series of thermogravimetric analyses combined with infrared detection (TGA-FTIR) of released gases was performed separately for silica, SiO_2_–PHMG, SiO_2_–PHMG–NRS, and SiO_2_–PHMG–NNS samples. For this purpose all samples were analysed at heating up to 760°С in the air flow. No gas release was noted for analysis of silica except for small amount of water vapours. TGA of SiO_2_-PHMG showed one-step decomposition of PHMG layer with release of ammonia and cyclohexane, while maximum decomposition rate was observed at 257°С.

**Figure 4:**
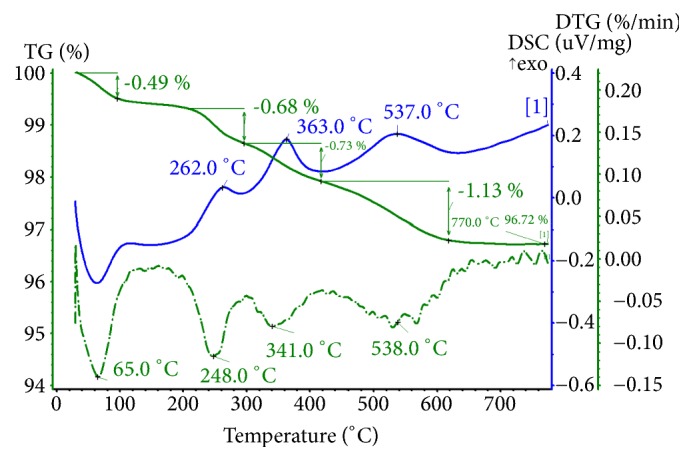
Data on total (solid dark line, TG, %) and differential mass (solid light line, DTG, %/min) changes of SiO_2_–PHMG–NNS obtained at heat rate 10 К/min at the air flow rate 50 mL/min. DSC profile is shown by dashed line. Similar dependences were observed during TGA-FTIR analysis of SiO_2_–PHMG–NRS.

**Figure 5:**
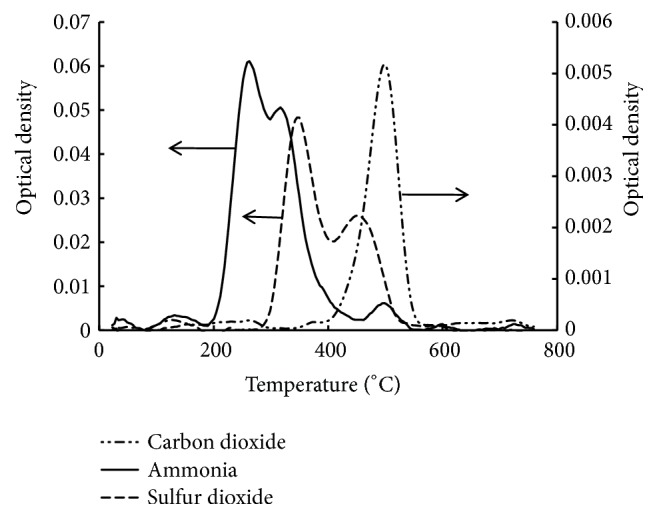
Temperature profiles of gases released during heating of SiO_2_–PHMG–NNS as measured by FTIR.

A more complex decomposition occurred in the case of final adsorbents. Figures 4 and 5 show TGA and profiles of released decomposition gases for adsorbent SiO_2_–PHMG–NNS. There are three steps in the decomposition process, which are related to the corresponding maxima in differential TGA curve. As mentioned above maximum at 262°С corresponds to decomposition of PHMG layer (Figure 4). Decomposition of adsorbed NNS occurred at 363°С with release of SO_2_ due to transformation of sulpho groups followed by oxidation of hydrocarbon residue with release of СО_2_ at 537°С (Figure 5). These data are confirmed by DSC profile (Figure 4).

### 3.3. Adsorption of Metal Ions under Static Conditions

#### 3.3.1. Effect of* pH*

**Figure 6:**
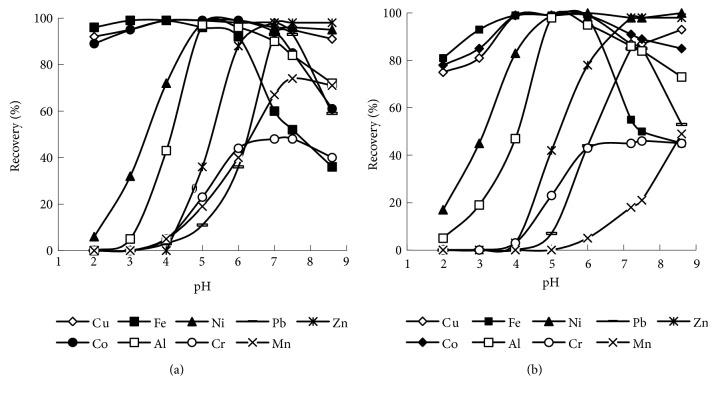
Effect of pH on adsorption of 0.2 *μ*g mL^−1^ Cu(II), Fe(III), Co(II), Ni(II), Al(III), Zn(II), Pb(II), Mn(II), and Cr(III) on SiO_2_–PHMG–NRS (a) and on SiO_2_–PHMG–NNS (b). Mass of adsorbent-0.1 g, sample volume 10 mL, and shaking time 10 min.

The adsorption of transition metal cations was studied with complexing adsorbents SiO_2_–PHMG–NRS and SiO_2_–PHMG–NNS having different position of nitroso and naphthol groups in the molecules and different number of sulpho groups. It should be noted that due to the different charge of the molecules the concentration of adsorbed NNS in SiO_2_–PHMG–NNS is twice higher than concentration of NRS in SiO_2_–PHMG–NRS. Obviously, both factors can influence the adsorption of metal ions. The adsorption of Cu(II), Fe(III), Co(II), Ni(II), Al(III), Zn(II), Pb(II), Mn(II), and Cr(III) was studied under static conditions from the aqueous solutions containing all metals in pH range from 2.0 to 8.5. It was found that adsorbent SiO_2_–PHMG–NRS extracted quantitatively (≥ 98%) Fe(III) at рН> 2, Cu(II) and Co(II) at рН ≥ 4.0, Ni(II) and Al(III) at рН ≥ 5, and Zn(II) at рН ≥ 7 as shown in Figure 6(a). Maximum extraction of Pb(II) and Mn(II) of 85 и 70%, respectively, was achieved at рН > 7, and adsorption of Cr(III) did not exceed 45% at рН 6 – 8.

Similar dependences of recovery of Cu(II), Fe(III), Co(II), Ni(II), Al(III), Zn(II), Pb(II), Mn(II), and Cr(III) on рН were obtained for the adsorbent SiO_2_–PHMG–NNS (Figure 6(b)). The lower recovery values of Cu(II), Fe(III), and Co(II) at pH 2 - 3 were observed as compared with data obtained with SiO_2_–PHMG–NNS, which is more stable under acidic conditions (see Figure 1). In general the pH dependences are in agreement with stability constants of metals complexes with sulphonated nitrosonaphthols shown in Table 4.

#### 3.3.2. Effect of Time

The effect of shaking time (varied from 2 to 40 min) on adsorption of seven metal cations was studied under static conditions. It was found that agitation of sample with adsorbent for 3 min is sufficient to achieve maximum recovery (> 98%) of Fe(III), Co(II), and Al(III). Quantitative recoveries of Cu(II) and Ni(II) required 5 min, and 10 min was required for complete recovery of Zn(II) and Pb(II). These results prove the excellent kinetics of adsorption of studied metal ions on the adsorbents SiO_2_–PHMG–NRS and SiO_2_–PHMG–NNS. At pH 7.0 the optimum adsorption time for all studied metals was achieved after 10 min and this time was used in further experiments.

#### 3.3.3. Adsorption Capacity

**Figure 7:**
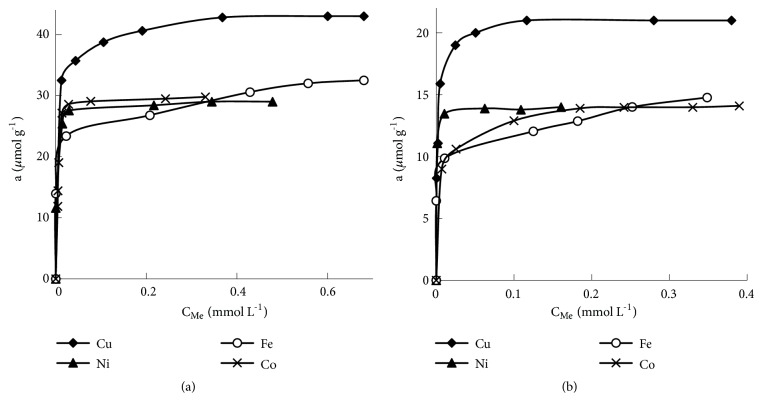
Adsorption capacity of Cu(II), Ni(II), Fe(III), and Co(II) on SiO_2_-PHMG-NRS* (a)* and SiO_2_-PHMG-NNS* (b). *Mass of adsorbent-0.1 g, sample volume 10 mL, and shaking time 10 min.

The adsorption capacity is an important characteristic of adsorbent showing how many adsorbents are required for the removal of specific amount of metal ions from the solutions. The adsorption capacity was measured for Cu(II), Ni(II), Fe(III), and Co(II) containing samples according to procedure described in Section 2.4. The dependences of adsorption of metals (*μ*mol g^−1^)* versus* the concentration of metal ions (*μ*mol L^−1^) are presented in Figure 7. According to the obtained results, the SiO_2_-PHMG-NRS can extract 21.0 *μ*mol g^−1^ of Cu(II), 13.8 *μ*mol g^−1^ of Ni(II), 14.7 *μ*mol g^−1^ of Fe(III), and 14.1 *μ*mol g^−1^ of Co(II) jointly presented in the sample. The adsorption capacity of SiO_2_-PHMG-NNS for Cu(II), Ni(II), Fe(III), and Co(II) was 42.7, 29.0, 32.5, and 29.8 *μ*mol g^−1^, respectively. Obviously, SiO_2_-PHMG-NNS has a much higher adsorption capacity for metal ions, which was related to the higher concentration NNS on surface of SiO_2_-PHMG (88 *μ*mol g^−1^).

### 3.4. Preconcentration of Metal Ions in Dynamic Mode

#### 3.4.1. Effect of* pH*

The adsorption of Cu(II), Fe(III), Co(II), Ni(II), Al(III), Zn(II), and Pb(II) was also studied under dynamic condition according to procedure described in Section 2.5. There were no observed changes in pH ranges of quantitative recovery (> 98%) of Fe(III), Co(II), and Al(III), but these pH ranges were broader and shifted to acidic region with рН 3 – 7, 4 – 7.5, and 6.5 – 8.0 for Cu(II), Ni(II), and Zn(II), respectively. The complete adsorption of Pb(II) was achieved at pH 6.5; therefore this value was selected as the optimum for column procedure.

#### 3.4.2. Effect of the Flow Rate

The increase of sample flow rate allowed significant decrease of time required for the analysis of the samples of big volume. The effect of the flow rate on retention of the studied ions was investigated under the optimum conditions. The flow rates were varied in the range of 0.5 – 6.0 mL min^−1^. It was found that the recoveries of Cu(II), Fe(III), Co(II), and Al(III) on SiO_2_-PHMG-NRS and SiO_2_-PHMG-NNS were practically not changed up to the flow rate of 5 mL min^−1^. The increase of flow rate to 2.5 mL min^−1^ decreased recovery of Ni(II) to 90%. At the same time, the recoveries Zn(II) and Pb(II) decreased significantly at flow rates higher than 1.5 mL min^−1^. Based on these results, 1.5 mL min^−1^ was selected as optimum flow rate for column preconcentration procedure.

#### 3.4.3. Effect of Sample Volume

**Figure 8:**
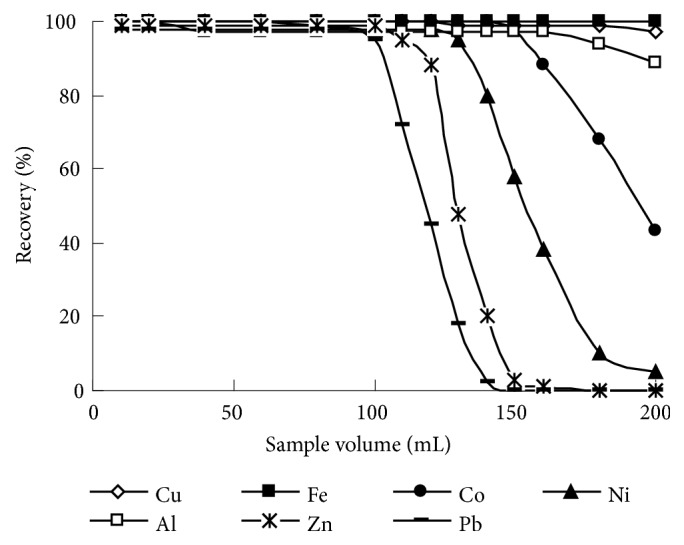
The effect of sample volume on metal recoveries for SiO_2_-PHMG-NRS. Mass of adsorbent in the column 0.1 g, рН 6.5, and flow rate 1.5 mL min^−1^. Concentration of metals: 200 ng mL^−1^ of Fe(III), 50 ng mL^−1^ of Cu(II), Al(III), Zn(II), and 20 ng mL^−1^ of Co(II), Ni(II), Pb(II).

The efficient preconcentration of metals from big sample volumes is vital for their determination in waters at trace levels. At the same time the concentration of certain metals, for example, iron, could be significantly higher than other metals considered in this work. On this reason a model solution containing 200 ng mL^−1^ of Fe(III), 50 ng mL^−1^ each of Cu(II), Al(III), and Zn(II), and 20 ng mL^−1^ each of Co(II), Ni(II), and Pb(II) with pH 6.5 was used for the investigation of the recovery of metals as a function of sample volume passed through the column packed with 0.1 g of either SiO_2_-PHMG-NRS or SiO_2_ - PHMG-NNS at flow rate 1.5 mL min^−1^. The concentration of metals was determined in every 10 mL portion of the effluent by ICP-OES. The results obtained for column packed with SiO_2_-PHMG-NRS are shown in Figure 8. The complete extraction of all metal cations was achieved for sample volumes less than 100 mL. An increase of model solution volumes up to 120 mL results in column breakthrough with recovery of Pb(II) and Zn(II) at the levels 45 and 88%, respectively. After passing 150 mL of model solution no more extraction was observed for Pb(II) and Zn(II), while recovery of Ni(II) decreases to 58%. Finally, 200 mL of model sample solution was loaded and a complete recovery (> 98%) was obtained only for Cu(II) and Fe(III). The observed extraction efficiency is in good agreement with values of stability of complexes of studied metals with NRS.

It was found that the column packed with 0.2 g SiO_2_-PHMG-NRS can provide complete recovery of all metals from 200 mL of model solutions. Certainly, for analysis of relatively pure natural waters such as mineral, tap, and mountain river water the sample volume can be also increased. The increased volume 400 mL of model solution was passed through the column packed with SiO_2_-PHMG-NNS. A complete recovery of Cu(II) and Fe(III) was observed in the whole volume of passed sample, of Al(III), Co(II), and Pb(II) in 360 mL, of Сo(II) in 280 mL, and of Pb(II) and Zn(II) in 200 mL. Compared with SiO_2_-PHMG-NRS the maximum volume of model solution passed through the column containing 0.1 g of SiO_2_-PHMG-NNS with complete extraction of all metals is increased to 200 mL. Obviously, this is due to the higher reagent loading in the case of SiO_2_-PHMG-NNS.

### 3.5. Desorption of Metals

The efficacy of complexing adsorbents in analysis of waters by ICP-OES depends on both quantitative adsorption of metals from the diluted solution and complete desorption of adsorbed metals in the smallest possible volume. The desorption of metals from SiO_2_-PHMG-NNS and SiO_2_-PHMG-NRS was studied by using 10 mL of HCl and HNO_3_ of concentration varied from 0.1 to 6 M. It was demonstrated that desorption degree of Cu(II), Fe(III), Ni(II), Al(III), Zn(II), and Pb(II) under static conditions does not depend on type of acid and reached maximum with 1 М solutions of HNO_3_ (Table 5).

Under dynamic conditions the desorption of metal ions was studied using column filled with 0.1 g of SiO_2_-PHMG-NRS or SiO_2_-PHMG-NNS adsorbents and 5 or 10 mL of 1 M HNO_3_ as eluent. The results showed that the quantitative desorption of Cu(II), Fe(III), Ni (II), Al(III), Zn(II), and Pb(II) was achieved by passing 5 ml of 1 M HNO_3_ at flow rate of 1 mL min^−1^ or 10 mL of 1 M HNO_3_ at flow rate of 2 mL min^−1^. Some part of loaded reagents is desorbed together with metals. However, the low concentration of the organic reagents in the eluate does not affect the ICP-OES determination of metals.

Quantitative desorption of Co(II) from the surface of adsorbents was not achieved either in static or in dynamic mode even with 6 M acids. The adsorbents remain colored red, which is characteristic for [CoL_3_]^6-^ complexes (where L is NRS or NNS), even in strongly acidic media. In this case, the Co concentration can be determined by a sorption-photometric method directly in the phase of the adsorbent using diffuse reflectance spectroscopy as described in [31].

### 3.6. Analytical Performance

As mentioned above the complete desorption of Сo(II) cannot be achieved with 1 М HNO_3_ as effluent, so determination of Cu(II), Fe(III), Ni(II), Al(III), Zn(II), and Pb(II) was developed. The LODs values calculated using 3*s *values for 10 blank measurements and preconcentration factors (PF) and RSD are presented in Table 6.

The relative standard deviations (RSD) were calculated from the data obtained in five parallel adsorption-desorption experiments for each of the studied metal ions. The results showed that the RSD values of the method are lower than 2.5% and the method has good precision for the analysis of trace Cu(II), Fe(III), Ni(II), Al(III), Zn(II), and Pb(II) from solution samples.

The results of the comparison of the analytical performance of the developed method with other methods reported previously are shown in Table 7. As seen, LOD values obtained by this method are not inferior to those published earlier using different adsorbents. It should be noted that the LODs of elements decrease proportionally to the increase of the ratio of the sample volume to the volume of eluent used for desorption. In turn, the required sample volume depends on the concentration of elements to be determined, as well as on the adsorption capacity of the adsorbent used for preconcentration. Obviously, the larger sample volumes can be used for the adsorbents with higher adsorption capacity that also decreases LOD values.

It should be also noted that the mechanical strength of strong base anion-exchange polymer resins is not too high due to osmotic pressure observed for resin with quick changes in ionic strength and acid concentrations. Also, the preparation of polymer resin based adsorbents as well as nanocarbon containing adsorbents is significantly more expensive as compared to silica modified with organic reagents. Among silica based adsorbents included in Table 7 SiO_2_-PHMG-NNS demonstrated better selectivity towards many metal cations. Again, SiO_2_-PHMG-NNS was obtained by simple soaking (physical adsorption) of silica in aqueous solutions of PHMG and sulphonated nitrosonaphthols, and preparation of other silica based adsorbents required more complex treatment with functionalized silanes in organic solvents.

### 3.7. Effect of Sample Matrix

Normally natural waters contain substantial amounts of inorganic anions including chlorides, nitrates, sulphates, carbonates, and alkali and alkaline-earth metal cations, which can potentially influence adsorption of Cu(II), Fe(III), Ni(II), Al(III), Zn(II), and Pb(II) on studied adsorbents. A possible effect of excessive concentration of these ions on recovery of transition metals from solutions was studied. It was found that alkali (Na^+^, K^+^) and alkaline-earth (Sr^2+^, Ca^2+^, Mg^2+^, and Ba2^+^) metal cations are not adsorbed by SiO_2_-PHMG-NRS or SiO_2_-PHMG-NNS from aqueous solutions with рН 6.5. Also, the presence of up to 10 mg L^−1^ of Сr(III), Cd(II), and Mn(II) in the samples does not interfere with the recovery of Cu(II), Fe(III), Ni(II), Al(III), Zn(II), and Pb(II). These metals can be quantitatively extracted from the solutions (up to 5 g L^−1^) of NaCl, NaNO_3_, NaHCO_3_, and Na_2_SO_4_, while a partial desorption of metals in a form of complexes with NRS and NNS was observed with further increase of salts concentrations to 10 g L^−1^ in the sample of 50 mL volume passed through the column.

### 3.8. Analysis of Natural Waters

The developed method was applied for ICP-OES determination of Cu(II), Fe(III), Ni(II), Al(III), Zn(II), and Pb(II) in natural waters. The samples included mineral waters Bay-Khaak (sample 1, Tuva Republic, Russia) and Arzhaan-Suu (sample 2, Altai Republic, Russia) and river water (sample 3), collected in estuarine of US river (Krasnoyarsk region, Russia). Adsorbent SiO_2_-PHMG-NRS was used for analysis of samples 1 and 2, and adsorbent SiO_2_-PHMG-NNS was used for analysis of samples 2 and 3. The analytical results for each of the environmental water samples, including recoveries for spiked samples (standard addition calibration was used), are presented in Table 8. Data reported is the average of three separate analyses.

There is limited and controversial data in the literature regarding chemical composition of Arzhaan-Suu natural waters [43]. Arzhaan-Suu spring water is related to the groups of weakly alkaline (pH 8.6-9.0) hydrocarbonate magnesium-calcium containing mineral waters. The mineralisation degree of this water is 0.30-0.33 g L^−1^ and water hardness is moderate of 3.45–3.75 mg L^−1^ [40]. Additionally, average concentrations of 0.08-0.38 mg L^−1^ of fluorine, 3.0 *μ*g L^−1^ of selenium, 0.9-1.5 *μ*g L^−1^ of silver, 0.1 *μ*g L^−1^ of cadmium, 0.1 *μ*g L^−1^ of mercury, 18 *μ*g L^−1^ of lead, 2.4 mg L^−1^ of silica, 90 *μ*g L^−1^ of manganese, 70 *μ*g L^−1^ of total iron, 8 *μ*g L^−1^ of total chromium, 67 *μ*g L^−1^ of calcium, and 28 *μ*g L^−1^ of magnesium have been reported for this water. The results obtained in the present work (Table 8) are in good agreement with previously reported data. The added-found experiment is accomplished to validate the method.

## 4. Conclusions

A simple two-step method for the preparation of complexing adsorbents with nitrosonaphthol functional groups is developed. The first step included preparation of anion-exchanger by coating of silica surface with strongly adsorbed cationic polymer PHMG. Three different sulphonated nitrosonaphthols electrostatically retained by PHMG coated silica provided similar complexing properties for the selected transition metals. Interestingly, the adsorbents containing a weaker retained nitroso-N-salt exhibited the highest capacity for metal ions and the better preconcentration factors (up to 80). The adsorbents can be used for preconcentration of metals in analysis of natural waters with low concentrations of target metal ions. Fast adsorption kinetics of metals on SiO_2_–PHMG–NRS and SiO_2_–PHMG–NNS makes these adsorbents useful for possible applications as stationary phases in column liquid chromatography and flow injection analysis.

## Figures and Tables

**Table 1:** Wavelength and operating conditions used for ICP-OES determination of metals.

RF power, kW	1.3
Plasma gas flow, L min^−1^	15
Auxiliary Ar, L min^−1^	0.2
Nebulizer Ar, L min^−1^	0.8
Pump rate, mL min^−1^	1.5
Readings/replicate	3
Wavelength, nm	Cu: 324.752; Fe: 238.204; Co: 238.892; Ni: 231.604; Al: 308.215; Zn: 213.857;
	Pb: 220.353; Mn: 257.610; Cr: 267.709

**Table 2:** Desorption degree (*R*_desorb_) NRS, NKS, and NNS from the surface of SiO_2_-PHMG by washing with 10 mL of NaCl solution (mass of adsorbents 0.1 g, reagents content 40 *µ*Mg^−1^, *n* = 5, *P* = 0.95).

*C*_NaCl_, M	*R*_desorb_, %		
	NRS	NKS	NNS
0.05	1.5	1.0	6
0.1	12	13	17
0.25	15	15	20
0.5	18	19	27
1.5	56	55	61

**Table 3:** Desorption degree of reagents NRS, NKS, and NNS from the surface of SiO_2_-PHMG by washing with 10 mL of HNO_3_ or HCl solutions (mass of adsorbents 0.1 g, reagents content 40 *µ*Mg^−1^, *n* = 5, *P*=0.95).

*C*_acid_, M	*R*_desorb_, %					
	HNO_3_			HCl		
	NRS	NKS	NNS	NRS	NKS	NNS
0.001	0.2	0.2	10	0.4	0.5	6.5
0.01	11	10	50	20	20	45
0.1	30	29	71	46	45	77
1	77	77	81	84	84	86
6	88	90	90	88	88	90
6^*a*^	98	98	97	96	98	97

^*a*^–desorption with two volumes of hot (50°С) 6 М acid

**Table 4:** pKa values of 1-nitroso-2-naphthol-3,6-disufonic acid and 2-nitroso-1-naphthol-4-sufonic acids and stability of their complexes with transition metals (25°C, *μ* = 0.1) [32].

	1-Nitroso-2-naphthol-3,6-disufonic acid			2-Nitroso-1-naphthol-4-sufonic acid		
	p*K*_a1_ = 6.87			p*K*_a1_ = 6.15		
Metal	log⁡*β*_1_	log⁡*β*_2_	log⁡*β*_3_	log⁡*β*_1_	log⁡*β*_2_	log⁡*β*_3_
Mn(II)	2.68			2.19^* a*^		
Cd(II)	3.42	6.00		2.45		
Zn(II)	4.46	7.10		3.15		
Pb(II)	4.64	7.83		3.94		
Co(II)	5.40			4.34		
Ni(II)	6.90	12.5	17.3	5.50	10.42	14.45
Cu(II)	9.90^*a*^	15.6 ^*a*^		7.90 ^*a*^	13.10 ^*a*^	

^*a*^ – *μ* = 0

**Table 5:** Elution (%) of metal ions adsorbed on SiO_2_-PHMG-NRS and SiO_2_-PHMG-NNS with nitric acid solutions (eluent volume is 10 mL).

Metal	SiO_2_-PHMG-NRS			SiO_2_-PHMG-NNS		
	0.1М HNO_3_	1М HNO_3_	2М HNO_3_	0.1М HNO_3_	1М HNO_3_	2М HNO_3_
Cu(II)	50.3	99.5	99.8	65.2	99.4	99.9
Fe(III)	30.8	99.5	99.9	37.5	99.7	99.8
Co(II)	-	8.5	12.0	-	8.3	10.7
Al(III)	78.5	99.9	99.9	85.9	99.6	99.8
Ni(II)	68.2	99.3	99.9	70.4	99.8	99.9
Zn(II)	83.4	99.9	99.8	89.4	99.8	99.8
Pb(II)	88.8	99.2	99.5	90.6	99.5	99.5

**Table 6:** LOD values of ICP-OES analysis and preconcentration factors (PF) obtained for selected metal ions with different adsorbents.

Metal	SiO_2_-PHMG-NRS			SiO_2_-PHMG-NNS		
	LOD, ng mL^−1^	PF	RSD, %	LOD, ng mL^−1^	PF	RSD, %
Cu(II)	0.76	40	0.68	0.75	80	0.77
Fe(III)	0.83	40	1.47	0.82	80	1.35
Al(III)	1.34	32	2.17	1.35	66	2.23
Ni(II)	0.95	26	1.64	0.96	28	1.52
Zn(II)	0.77	24	0.89	0.77	30	0.93
Pb(II)	0.87	20	2.35	0.88	42	1.98

**Table 7:** Comparison of analytical characteristics of the preconcentration methods for determination of elements in water samples.

Adsorbent/chelating agent	Method	Element - LOD (*µ*g L^−1^)	Eluent	PF	Ref.
Amberlite XAD-4/salicylaldehyde benzoylhydrazone	FAAS	Cu(II) - 0.50, Ni(II) - 0.90, Co(II) - 0.70, Fe(III) - 0.40	10 mL 3 M HNO_3_ in acetone	240	[33]
Amberlite XAD-16/p-aminobenzenesulfonic acid	FAAS	Cu(II) - 0.72, Ni(II) - 0.89, Zn(II) - 1.05, Co(II) - 0.98, Cr(III) - 1.17, Fe(III) - 0.69, Pb(II) – 1.91	3-5 mL 2 M HNO_3_	60-100	[34]
Amberlite XAD-4/1-(2-pyridylazo)-2-naphthol	FAAS	Co(II) - 0.54, Ni(II) - 1.30, Mn(II) - 0.20, Zn(II) - 0.28, Pb(II) - 1.10, Cu(II) - 0.42	5 mL 2 M HClO_4_ for Cu(II), Ni(II); 5 mL 2 M HNO_3_ for Zn(II), Co(II); 2.5 mL 0.5 M H_2_SO_4_ for Cd(II), Pb(II) and Mn(II)	160-400*∗*	[35]
Chromosorb 106-TAR/4-(2-thiazolylazo) resorcinol	ICP-MS	Cu(II) - 0.012, Ni(II) - 0.0064 Pb(II) - 0.021, Zn(II) - 0.015	1 mL, 0.75 M HNO_3_	5	[36]
Silica/NH_2_	ICP-MS	Pb(II) - 0.699, Cd(II) - 0.123, Zn(II) - 0.24	3 mL, 3M HNO_3_	33.3	[37]
Silica/N-(2-aminoethyl)-2,3-dihydboxybenzaldimine	ICP-OES	Cd(II) – 0.012, Cu(II) - 0.098 Ni(II) - 0.056, Pb(II) - 0.14	5 mL, 0.5 M HCl	100	[38]
Silica/sodium diethyldithiocarbamate	ICP-OES	Co(II) – 0.198, Ni(II) – 0.201	5 m, 2 MHNO_3_	400	[39]
GO Amberlite XAD-16/picolylamine	ICP-OES	Cu(II) – 0.048, Pb(II) – 1.43	5 mL, 2 МHCl	150	[40]
Multiwall carbon nanotubes /silica	ICP-OES	As(V) – 0.67, Cd(II) – 0.45, Cu(II) - 0.11, Cr(III) – 0.91, V(V) – 0.55, Zn(II) – 0.27	0.6 mL, 2 МHCl	10	[41]
SiO_2_-PHMG-NNS	ICP-OES	Cu(II) - 0.75, Fe(III) - 0.82, Al(III) - 1.35, Ni(II) - 0.96, Zn(II) - 0.77, Pb(II) - 0.88	5 mL, 1 МHNO_3_	28-80	This work

*∗* - for 0.5 mg of sorbent

**Table 8:** The results of ICP–OES determination of metals in natural waters (sample volume 100 mL acidified with 5 mL of 1 М HNO_3_).

Metal	SiO_2_-PHMG-NRS				SiO_2_-PHMG-NNS			
	Added, ng mL^−1^	Found, ng mL^−1^	Added, ng mL^−1^	Found, ng mL^−1^	Added, ng mL^−1^	Found, ng mL^−1^	Added, ng mL^−1^	Found, ng mL^−1^
	Sample 1		Sample 2		Sample 2		Sample 3	
Cu(II)	- 5.0	0.34±0.01 5.37±0.05^*a*^	- 5.0	2.32±0.04 7.33±0.07	- 5.0	2.28±0.04 7.26±0.07	- 5.0	0.97±0.01 5.99±0.06
Fe(III)	- 5.0	0.72±0.01 5.72±0.05	- 5.0	1.69±0.03 6.65±0.06	- 5.0	1.71±0.03 6.69±0.06	- 5.0	10.7±0.1 15.7±0.1
Ni(II)	- 2.0	0.21±0.01 2.19±0.03	- 2.0	0.60±0.02 2.58±0.03	- 2.0	0.56±0.02 2.57±0.03	- 2.0	2.85±0.04 4.82±0.04
Al(III)	- 2.0	2.44±0.04 4.43±0.04	- 2.0	4.02±0.04 6.01±0.06	- 2.0	3.99±0.04 6.02±0.06	- 2.0	7.33±0.08 9.40±0.10
Zn(II)	- 2.0	1.62±0.03 3.60±0.04	- 2.0	2.56±0.04 4.52±0.05	- 2.0	2.49±0.04 4.52±0.05	- 2.0	5.05±0.05 7.02±0.07
Pb(II)	-	ND^*b*^	-	0.20±0. 02	-	0.22±0.02	-	0.48±0.03
	2.0	2.03±0.04	2.0	2.23±0.04	2.0	2.24±0.04	2.0	2.50±0.04

${}^{a}\;\;\overset{-}{x}\pm s$ (*n* = 5), $\overset{-}{x}$- average for five determinations, *s* - standard deviation, ^*b*^ND – non-detected.

## Data Availability

The data used to support the findings of this study are included within the article.
